# The Associations between Evacuation Status and Lifestyle-Related Diseases in Fukushima after the Great East Japan Earthquake: The Fukushima Health Management Survey

**DOI:** 10.3390/ijerph19095661

**Published:** 2022-05-06

**Authors:** Zhichao Sun, Hironori Imano, Eri Eguchi, Fumikazu Hayashi, Tetsuya Ohira, Renzhe Cui, Seiji Yasumura, Akira Sakai, Michio Shimabukuro, Hitoshi Ohto, Kenji Kamiya, Hiroyasu Iso

**Affiliations:** 1 Public Health, Department of Social Medicine, Osaka University Graduate School of Medicine, Osaka 565-0871, Japan; szcsun@pbhel.med.osaka-u.ac.jp (Z.S.); imano@pbhel.med.osaka-u.ac.jp (H.I.); 2 Health Town Development Science Center, Yao City Health Center, Osaka 581-0006, Japan; 3 Department of Public Health, Kindai University Faculty of Medicine, Osakasayama 589-8511, Japan; 4 Department of Epidemiology, Fukushima Medical University School of Medicine, Fukushima 960-1295, Japan; e-eguchi@fmu.ac.jp (E.E.); fhayashi@fmu.ac.jp (F.H.); teoohira@fmu.ac.jp (T.O.); 5 Radiation Medical Science Center for the Fukushima Health Management Survey, Fukushima Medical University, Fukushima 960-1295, Japan; yasumura@fmu.ac.jp (S.Y.); sakira@fmu.ac.jp (A.S.); mshimabukuro-ur@umin.ac.jp (M.S.); hit-ohto@fmu.ac.jp (H.O.); kkamiya@hiroshima-u.ac.jp (K.K.); 6 Department of Internal Medicine, Okanami General Hospital, Iga 518-0842, Japan; cuirenzhe@hotmail.com; 7 Department of Public Health, Fukushima Medical University School of Medicine, Fukushima 960-1295, Japan; 8 Department of Radiation Life Sciences, Fukushima Medical University School of Medicine, Fukushima 960-1295, Japan; 9 Department of Diabetes, Endocrinology and Metabolism, Fukushima Medical University School of Medicine, Fukushima 960-1295, Japan; 10 Research Institute for Radiation Biology and Medicine, Hiroshima University, Hiroshima 734-8553, Japan; 11 Institute for Global Health Policy Research, National Center for Global Health and Medicine, Tokyo 162-8655, Japan

**Keywords:** evacuation, Great East Japan earthquake, disaster, disease prevalence status, cardiovascular and metabolic diseases

## Abstract

Background: This study aimed to investigate the association between evacuation status and lifestyle-related disease risks among Fukushima residents following the Great East Japan earthquake. Methods: Fukushima health management survey respondents were classified into non-evacuees, returnees, evacuees in lifted areas, and evacuees in banned areas. During a seven-year follow-up, 22,234 men and 31,158 women were included. Those with a history of diabetes, hypertension, or dyslipidemia at baseline were excluded. The odds ratios of risk factors (ORs) and 95% confidence intervals (CIs) for diabetes, hypertension, and dyslipidemia were calculated using a logistic regression model. Spatial autocorrelation of the prevalence of these diseases in the Fukushima area in 2017, was calculated to detect the disease prevalence status. Results: The risks of diabetes, hypertension, and dyslipidemia were higher in evacuees in banned areas than in non-evacuees; the multivariable ORs were 1.32 (95% CI: 1.19–1.46), 1.15 (1.06–1.25), and 1.20 (1.11–1.30) for diabetes, hypertension, and dyslipidemia, respectively. Returnees and evacuees in lifted areas had no increased risk of diseases. The area analyzed had a non-uniform spatial distribution of diabetes, hypertension, and hyperlipidemia, with clusters around Fukushima and Koriyama. Conclusion: Our findings imply the need for continuous support for evacuees in banned areas.

## 1. Introduction

The Great East Japan earthquake occurred on 11 March 2011, causing a large tsunami [1] and a severe accident at the Fukushima Dai-ichi Nuclear Power Plant [2]. These serious disasters resulted in extensive damage to the coastal area adjacent to the east of Fukushima, infrastructure destruction, and potential ultra-low-dose level radioactive pollution. Thus, many residents needed to evacuate, as implemented by the national and Fukushima Prefecture governments [3].

Evacuation affects lifestyle and has been associated with increased alcohol consumption [4], high smoking prevalence [5], and impaired sleep quality [6]. Lifestyle changes, such as those mentioned above, have a strong effect on lifestyle-related diseases. Moreover, changes in the living environment and socio-economic factors [7,8] could affect the mental health of the evacuees. People who were forced to leave their homes were more likely to develop post-traumatic stress disorder [9,10], and approximately 4.7% of the residents in the Fukushima Prefecture lost or changed their job [11]. Previous studies have also shown that evacuees had higher risks of diabetes, heart disease, and sudden cardiac death [12,13] than non-evacuees.

To date, restrictions have been lifted in 67.8% of the previously restricted areas [14], and the national and prefectural governments have encouraged the evacuees to return to their homes. However, some people remained reluctant to return, although the areas were cleaned and declared safe [15]. Therefore, people who continued to evacuate have been forced to live in temporary houses and face new interpersonal relationships.

Evacuation status may impact lifestyle and cardiovascular risk factors, such as diabetes, hypertension, and dyslipidemia. In this study, we hypothesized that the evacuees forced to live outside their original houses in banned areas may have a higher risk of diabetes, hypertension, and dyslipidemia, and that returnees and evacuees in lifted areas do not have these increased risks. We used the database affiliated with the Fukushima health management survey to test this hypothesis.

## 2. Materials and Methods

### 2.1. Participants

We used the following three databases from the Fukushima health management survey (FHMS) in 2017 [16]: comprehensive health checks, mental health and lifestyle survey, and basic survey. Comprehensive health checks included two sets of respondents as follows: (1) people in the evacuation zone specified by the government and (2) people outside of the evacuation zone in the Fukushima Prefecture. The evacuation zone comprised Iitate Village (mura), Kawauchi Village, Katsurao Village, Hirono Town (machi), Naraha Town, Tomioka Town, Okuma Town, Futaba Town, Namie Town, Minamisoma City, and Tamura City. The mental health and lifestyle survey included these 13 areas.

**Figure 1:**
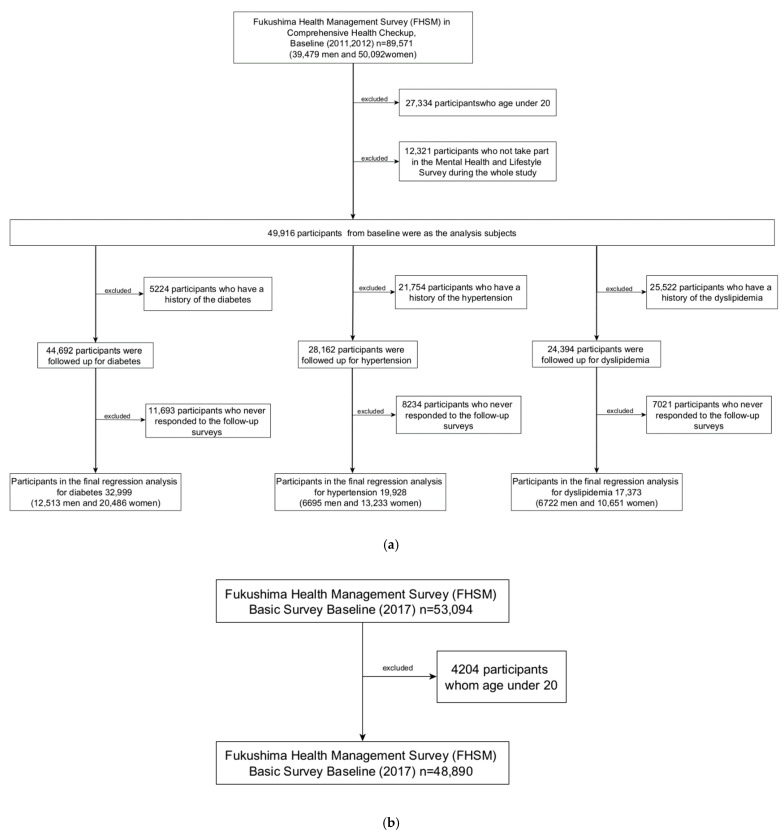
Flow diagram of the participant selection process: (**a**) longitudinal analysis; (**b**) spatial analysis.

Figure 1a presents a flow chart of the longitudinal analysis used in this study, with a follow-up for up to 7 years. Among the 89,571 participants of the comprehensive health check database, we excluded 27,334 who were aged <20 years and 12,321 who did not participate in the mental health and lifestyle survey. A total of 49,916 participants were included in the analysis. Subsequently, we excluded participants with a history of diabetes (*n* = 5224), hypertension (*n* = 21,754), or dyslipidemia (*n* = 25,522) at baseline. At follow-up, there were 11,693 participants with diabetes, 8234 with hypertension, and 7021 with dyslipidemia who never responded. Finally, we analyzed 32,999 participants with diabetes, 19,928 with hypertension, and 17,373 with hyperlipidemia.

For the spatial analysis, 53,094 individuals were included in the 2017 total comprehensive health check database. We excluded 4204 individuals aged <20 years. Finally, 48,890 individuals were included in the analysis (Figure 1b).

### 2.2. Changes in Evacuation Status

**Figure 2:**
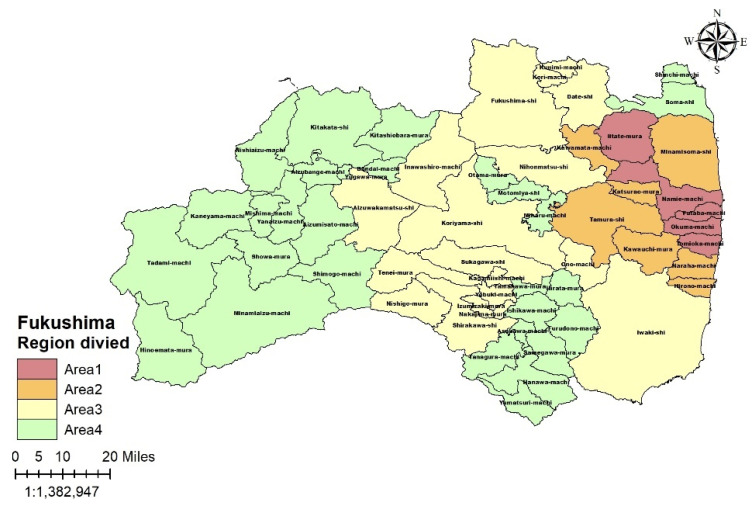
Group design based on the history of the Fukushima evacuation area and caution area. Area 1: still difficult to return at the time of the deadline; Area 2: where the evacuation alerts have been lifted at the time of the deadline; Area 3: near the evacuation area or with a history of voluntary evacuation; and Area 4: all other areas.

Figure 2 shows the changes in the evacuation areas in the Fukushima Prefecture in 2017, based on the information provided by the national and local governments [14,17]. As of 2017, areas that still have restrictions were labeled as Area1 (red color); those that have lifted restrictions, Area 2 (orange color); those with a history of voluntary refuge [17], Area 3 (yellow color); and those outside of the Fukushima Prefecture, Area 4 (green color).

Evacuees were defined as follows: those who had lived in Area 2 or 3 before the earthquake and evacuated from lifted areas until 2017 were defined as evacuees from lifted areas, and those who lived in Area 1 before the earthquake were defined as evacuees from banned areas. Non-evacuees were defined as all individuals living in Areas 3 and 4 who never changed their residences. Returnees were defined as individuals who lived in Area 2 before the earthquake, evacuated to Area 3 or 4 after the earthquake, and returned to their homes in Area 2 before 2017.

### 2.3. Lifestyle Behaviors and Social Factors

Smoking and drinking behaviors, sleep, physical activity, job change, and education level were obtained from the mental health & lifestyle survey data. We assessed the smoking status of the participants using the question, “Do you smoke?” with the following options: “non-smoker”, “ex-smoker”, and “current smoker”. Those who selected “current smoker” were considered as current smokers. Participants’ alcohol intake was assessed using the question, “Do you consume alcohol?” with the following options: “non-drinker (less than once per month)”, “ex-drinker”, and “drinker (once or more per month)”. Those who selected “drinker (once or more per month)” were considered as current drinkers. Sleep quality was evaluated using the question, “Are you satisfied with the length of sleep for the past month?” with the following options: “satisfied” and “not satisfied”. Physical activity level was assessed using the question, “Do you exercise regularly?” with the following options: “≥daily”, “2–4 times/week”, “weekly”, and “almost never”. Those who selected “≥daily”, “2–4 times/week”, or “weekly” were considered to have a physical activity frequency of at least once a week. Education level was assessed by the question, “What is your last educational level?” with the following options: “elementary or junior high school”, “high school”, “vocational school or junior college”, and “university or graduate school”. Those who selected “university or graduate school” were considered to have received college or higher education. Change of job was assessed by the question, “Did you experience a change in work situation since the disaster?” with the following options: “yes” and “no”. Psychological distress was evaluated using Kessler Psychological Distress (K6), and participants with a score of ≥13 were considered to have psychological distress.

Weight was measured in light indoor clothing without shoes, and height was recorded barefoot by well-trained staff. Weight and height measurements were obtained from comprehensive health check data. Body mass index (BMI) was calculated as weight (kg)/[height] (m)^2^.

### 2.4. Onset of Diabetes, Hypertension, and Dyslipidemia

The onset of diabetes mellitus, hypertension, and dyslipidemia was acquired from the comprehensive health check data. Hypertension was defined as systolic blood pressure (SBP) ≥ 140 mmHg, diastolic blood pressure (DBP) ≥ 90 mmHg [18], and/or the use of antihypertensive medication. Diabetes was defined as a fasting plasma glucose (FPG) level ≥ 126 mg/dL (7.0 mmol/L), random blood glucose (RBG) level ≥ 200 (11.1 mmol/L), HbA1c ≥ 6.5% [19], and/or the use of insulin injection or hypoglycemic drugs. Dyslipidemia was defined as plasma triglyceride (TG) level ≥ 150 mg/dL (fasting time), high-density lipoprotein cholesterol (HDL-C) level ≤ 40 mg/dL, low-density lipoprotein cholesterol (LDL-C) level ≥ 140 mg/dL [20], and/or the use of lipid-lowering agents.

### 2.5. Addresses and Standardized Prevalence Ratios in the Fukushima Prefecture

We used the current postal code from the basic survey data for the spatial analysis to ensure reliability. Diabetes, hypertension, and hyperlipidemia were defined based on the comprehensive health check database of the whole prefecture. The standardized prevalence ratios (SPRs) for diabetes, hypertension, and dyslipidemia were used to avoid distortion due to inappropriate age adjustment. The SPRs for diabetes, hypertension, and hyperlipidemia in each municipality in the Fukushima Prefecture were calculated compared to the 1985 Japanese standard population model. Municipality SPRs were calculated by dividing the municipality observed cases by the municipality expected cases [21,22].

### 2.6. Statistical Analysis

First, we calculated the age-adjusted mean values and prevalence of risk factors using analysis of covariance. Multiple linear regression was performed to compare the returnees, evacuees in lifted areas, and evacuees in banned areas with the non-evacuees.

Using the logistic regression model, age- and multivariable-adjusted odds ratios (ORs) and 95% confidence intervals (CIs) for diabetes, hypertension, and hyperlipidemia among the returnees, evacuees in lifted areas, and evacuees in banned areas, compared with the non-evacuees were calculated. The adjustment variables included age (continuous), BMI (quintiles), cigarette smoking status (never-smoker, ex-smoker, current smoker), alcohol consumption (non-drinker, ex-drinker, current drinker), physical activity (≥once weekly or <once weekly), sleep satisfaction (satisfied or not satisfied), change of job (yes or no), and educational status (elementary or junior high school, high school, vocational school or junior college, university or graduate school). Statistical analyses were conducted using SAS version 9.4 (SAS Institute, Inc., Cary, NC, USA). Two-tailed *p* values < 0.05 were considered statistically significant.

The global Moran’s index [23] was used to analyze regional spatial autocorrelation to identify geographic clustering. Hotspot analysis (Getis-Ord Gi*) [24] was used to determine the clusters. Hot spots represent a high-value spatial cluster of diabetes, hypertension, or dyslipidemia, whereas cold spots represent a low-value spatial cluster in the Fukushima Prefecture. Statistical significance was set at *p* < 0.05, and 90% CIs were dependent on the z < −1.65 or z > +1.65, whereas 95% CIs were dependent on the z < −1.96 or z > +1.96. All spatial analyses were conducted in ArcGis10.8.1 (Esri, Inc., Redlands, CA, USA).

## 3. Results

During a seven-year follow-up, 1822 participants had diabetes, 3609 had hypertension, and 4361 had dyslipidemia.

### 3.1. Characteristics of Participants at Baseline

Table 1 shows the age-adjusted mean values and characteristics at baseline according to the evacuation status. We found that 47.7% of the participants had been evacuated or were still evacuees. Compared with the non-evacuees, both evacuees in lifted areas and those in banned areas were younger and had a higher proportion of current smokers, current alcohol drinkers, dissatisfaction with sleep, change in their job, and university or graduate school education. Compared with the non-evacuees, the returnees were likely to have a lower average age and BMI and a higher proportion of dissatisfaction with sleep, change in their job, and university or graduate school education. Additionally, 11.6% of evacuees in banned areas had a K6 score of ≥13, which accounted for the highest proportion of individuals who had psychological distress.

### 3.2. Associations between Evacuate Status and Diabetes, Hypertension, and Dyslipidemia

Table 2 presents the age- and multivariable-adjusted odds ratios (ORs) and 95% confidence intervals (95% CIs) for diabetes, hypertension, and dyslipidemia for the returnees, evacuees in lifted areas, and evacuees in the banned areas. The ORs for diabetes, hypertension, and dyslipidemia for evacuees in the banned areas were significantly higher than those for non-evacuees, and these associations remained statistically significant even after adjusting for confounders. The multivariable ORs (95% CIs) were 1.35 (1.22–1.51) for diabetes, 1.14 (1.05–1.24) for hypertension, and 1.22 (1.13–1.32) for dyslipidemia. The ORs for diabetes, hypertension, and dyslipidemia were higher in returnees than that in non-evacuees, albeit not statistically significantly. There was no statistically significant association between the evacuees in lifted areas and the non-evacuees. With additional adjustment for psychological distress, the results still showed the same associations. Multivariable ORs (95% CIs) were 1.35 (1.21–1.50) for diabetes, 1.14 (1.05–1.24) for hypertension, and 1.22 (1.13–1.32) for dyslipidemia.

Gender-specific analyses (Table 3) showed similar associations, except for hypertension in men. The multivariable ORs (95% CI) for diabetes, hypertension, dyslipidemia were 1.33 (1.15–1.55), 1.08 (0.95–1.23), and 1.31 (1.16–1.48) among male evacuees in banned area and 1.38 (1.19–1.61), 1.20 (1.08–1.35), and 1.21 (1.09–1.34) among female evacuees. Additional adjustment for psychological distress also showed the same associations. Multivariable ORs (95% CIs) for diabetes, hypertension, and dyslipidemia were 1.33 (1.15–1.54), 1.08 (0.94–1.23), and 1.31 (1.16–1.48), respectively, among male evacuees in banned areas and 1.38 (1.18–1.60), 1.20 (1.08–1.35), and 1.20 (1.09–1.33) among female evacuees in banned areas.

### 3.3. Spatial Distribution Characteristics

**Figure 3:**
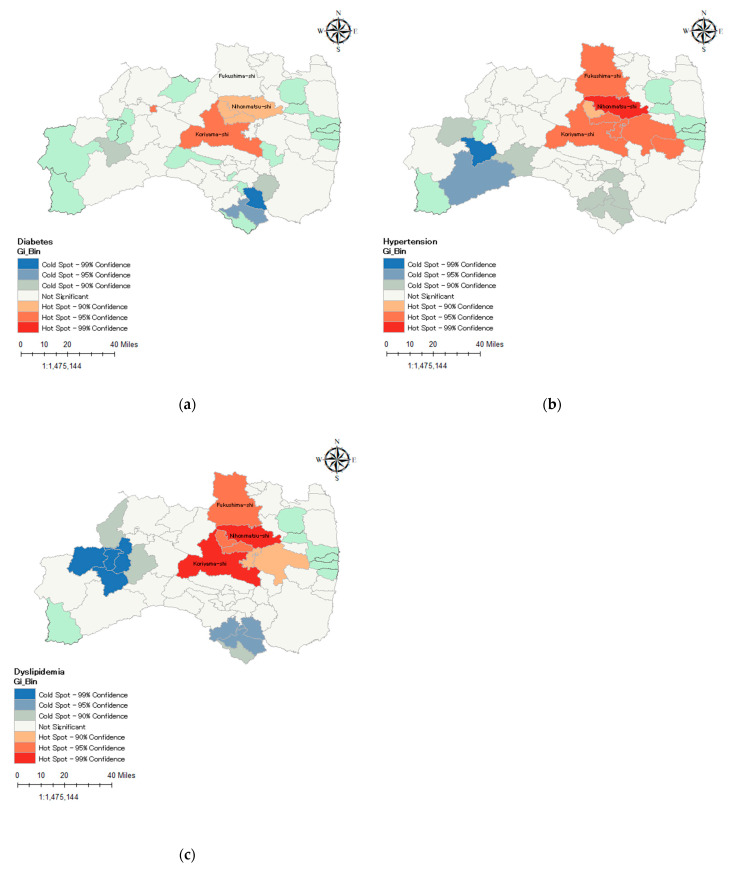
Hot spot analysis of spatial prevalence of lifestyle-related diseases among survey participants: (**a**) Spatial pattern of diabetes; (**b**) Spatial pattern of hypertension; (**c**) Spatial pattern of dyslipidemia.

The global spatial autocorrelation showed that the prevalence of diabetes, hypertension, and hyperlipidemia was positively spatially autocorrelated in Fukushima (Supplementary Table S1). The global Moran’s indexes for diabetes, hypertension, and dyslipidemia were 0.17, 0.16, and 0.34, respectively. The administrative region around the Fukushima and Koriyama cities were determined as clusters (Figure 3). However, Iwaki City is in the lower right corner of Fukushima Prefecture, so the spatial pattern may lack of significance.

## 4. Discussion

This study revealed that evacuees in banned areas had a higher risk of diabetes, hypertension, and dyslipidemia than non-evacuees, whereas returnees and evacuees in lifted areas did not have increased risks. These associations remained significant even after adjustment for selected lifestyles, education level, and change of job. Poor lifestyle factors including smoking, heavy alcohol consumption, physical inactivity, and inadequate sleep have been proven to enhance the incidence the lifestyle-related diseases [25,26]. Factors related to socioeconomic status such as low education level and change of job have also been confirmed as risk factors for the incidence of cardiovascular and metabolic diseases [7,27]. In addition, a high-high cluster of diabetes, hypertension, and dyslipidemia around the cities of Fukushima and Koriyama was noted. This study is the first to evaluate the risk of lifestyle-related diseases among returnees and evacuees in the lifted areas, and evacuees in the banned areas.

We attempted to explain why the evacuees in the banned areas had a higher risk of diabetes, hypertension, and hyperlipidemia than the other groups and the causes of spatial clustering in the discussion below.

First, in our study, the excess risks of diabetes, hypertension, and dyslipidemia among evacuees in banned areas were not altered after adjustment for psychological distress. However, this result in 2017 did not negate the possibility that that psychological distress confounded or mediated the excess risks probably because mental stress may temper over time [28].

Mental stress has been associated with an increased risks of diabetes [29], hypertension [30], and dyslipidemia [31,32]. Moreover, the incidence of diabetes increased [33,34] among evacuees immediately following the disaster. The hypothalamic–pituitary–adrenal axis [35,36] increases circulating cortisol levels, and under chronic stress conditions, the pituitary gland secretes vasopressin [35], which could affect glucose and lipid metabolism, leading to diabetes, hypertension, and dyslipidemia.

Second, diverse socio-economic factors may have influenced the incidence of lifestyle-related diseases. Evacuees in banned areas were closer to the center of the accident, were more vulnerable to the negative impact of the accident, and had no choice but to evacuate. Sugimoto et al. showed that long-term evacuation could lead to a poor perceived health status [35]. In addition, a recent report reported that evacuees in the banned area had less communication with others regarding their daily lives than those in the lifted areas [36]. These factors may have increased the risk of lifestyle-related disease onset.

Furthermore, the evacuees in the banned areas needed to leave their own houses and lose their material possessions and jobs, leading to a loss of purpose in life. Unemployment has been considered as a common factor that could increase the risk of delayed mental illness [37,38,39]. In addition, house damage, tsunami experience, nuclear power plant accident experience, and loss of family, realty, and close friends were associated with increased mental stress [40]. Moreover, we assumed that evacuees in the banned areas who were eager to return to their home but were unable to do so have a greater burden; thus, their risk of developing lifestyle-related diseases may be higher.

According to our findings, the prevalence clusters of hypertension, diabetes, and dyslipidemia were mainly located around the cities of Fukushima and Koriyama. Fukushima City is the provincial capital, whereas Koriyama City is one of the most populous commercial cities in the Fukushima province. Therefore, collective infrastructural resources are concentrated in Fukushima and Koriyama [41]. Additionally, after the disaster, these two cities, and the surrounding areas closest to the disaster site, quickly established emergency-relevant infrastructure and accepted many evacuees [42]. Therefore, this could partially explain why the spatial pattern of diabetes, hypertension, and dyslipidemia prevalence in the Fukushima and Koriyama cities were different from other cities.

Compared with other similar studies [13,43,44,45], this study has the following salient features. First, it analyzed a large population-based cohort, which not only included the residents in the affected areas of the Great East Japan earthquake, but also those throughout the entire Fukushima Prefecture. Second, over 70% of participants were followed-up for seven years from 2011–2017. Third, we adjusted for several potential confounders, including lifestyle and socioeconomic factors.

However, this study had some limitations. First, each participant may not have taken the comprehensive health checks and mental health and lifestyle surveys conducted annually. Therefore, we could not assess the impact of lifestyle changes on the incidence of diabetes, hypertension, and dyslipidemia. Second, we did not have data on the proportion of people who evacuated outside the Fukushima Prefecture and the prevalence of diseases in cities, towns, and villages in other prefectures around the Fukushima Prefecture. Third, regarding the spatial analysis, we only examined the prevalence of diabetes, hypertension, and dyslipidemia in 2017. Therefore, we could not examine the dynamic clustering process of each region. Lastly, the lifestyle parameters were based on a self-reported questionnaire and liable to misclassification.

Nevertheless, this is the first study to describe the prevalence and incidence of diabetes, hypertension, and dyslipidemia in the Fukushima area using both a cross-sectional design for the spatial dimension and a longitudinal design for the temporal dimension.

## 5. Conclusions

During a 7-year follow-up after the Great East Japan earthquake, evacuees in the banned areas had a higher incidence of diabetes, hypertension, and dyslipidemia than non-evacuees. Our findings imply the importance of continuous support for the prevention of lifestyle-related diseases for the evacuees in banned areas.

## Figures and Tables

**Table 1:** Characteristics of participants at baseline according to evacuation status (N = 49,916).

	Non-Evacuees (*n* = 26,115)	Returnees (*n* = 1573)	Evacuees in Lifted Areas (*n* = 5559)	Evacuees in Banned Areas (*n* = 16,669)
Age (years) Mean ± SD	57.4 ± 14.5	55.4 ± 14.1 ***	46.1 ± 16.5 ***	54.1 ± 15.2 ***
BMI (kg/m^2^) Mean ± SD	23.8 ± 3.77	23.7 ± 3.52	23.2 ± 3.89 ***	23.9 ± 3.92 ***
Current alcohol drinker (%)	34.9	38.2 **	38.5 **	37.9 ***
Current smoker (%)	13.1	12.8	16.7 **	16.6 ***
Sleep, inadequate (%)	26.4	33.4 ***	33.2 ***	32.8 ***
Physical activity, ≥ once/week (%)	40.8	40.2	32.7 **	39.7 ***
Change of job, yes (%)	33.9	56.5 ***	48.7 ***	53.5 ***
Education attainment, i.e., university or graduate school (%)	4.8	6.7 ***	11.1 ***	7.1 ***
Psychological distress (K6 score of ≥13) (%)	6.7	10.9 ***	9.4 ***	11.6 ***

Difference from non-evacuees: ** *p* < 0.01; *** *p* < 0.001.

**Table 2:** Age-adjusted and multivariable odds ratios of diabetes, hypertension, and dyslipidemia according to evacuation status.

	Total			
	Non-Evacuees	Returnees	Evacuees in Lifted Areas	Evacuees in Banned Areas
No. at risk, *n*	16,784	1284	3207	11,724
Diabetes, *n*	875	63	118	766
Age-adjusted OR (95% CI)	Ref.	1.04 (0.80–1.35)	0.96 (0.79–1.17)	1.45 (1.30–1.59) ***
Multivariable OR (95% CI) ^§^	Ref.	1.04 (0.79–1.35)	1.00 (0.82–1.22)	1.35 (1.22–1.51) ***
Multivariable OR (95% CI) ^§§^	Ref.	1.03 (0.79–1.35)	0.99 (0.81–1.21)	1.35 (1.21–1.50) ***
No. at risk, *n*	9367	808	2417	7336
Hypertension, *n*	1828	146	267	1368
Age-adjusted OR (95% CI)	Ref.	1.07 (0.88–1.29)	0.84 (0.73–0.97) *	1.17 (1.08–1.27) ***
Multivariable OR (95% CI) ^§^	Ref.	1.06 (0.87–1.29)	0.87 (0.75–1.00)	1.14 (1.05–1.24) **
Multivariable OR (95% CI) ^§§^	Ref.	1.06 (0.87–1.29)	0.87 (0.75–1.00)	1.14 (1.05–1.24) **
No. at risk, *n*	8628	617	2031	6097
Dyslipidemia, *n*	2100	152	421	1688
Age-adjusted OR (95% CI)	Ref.	1.10 (0.91–1.33)	0.97 (0.86–1.10)	1.28 (1.18–1.38) ***
Multivariable OR (95% CI) ^§^	Ref.	1.07 (0.88–1.30)	0.97 (0.86–1.10)	1.22 (1.13–1.32) ***
Multivariable OR (95% CI) ^§§^	Ref.	1.07 (0.88–1.29)	0.97 (0.86–1.10)	1.22 (1.13–1.32) ***

CI, confidence interval; OR, odds ratio. * *p* < 0.05; ** *p* < 0.01;*** *p* < 0.001. ^§^ Adjust for age, body mass index, smoking status, alcohol consumption, sports time, sleep quality, education level, and change of job. ^§§^ Adjusted further for psychological distress.

**Table 3:** Gender-specific age-adjusted and multivariable odds ratios of diabetes, hypertension, and dyslipidemia according to evacuation status.

	Men				Women			
	Non- Evacuees	Returnees	Evacuees in Lifted Areas	Evacuees in Banned Areas	Non- Evacuees	Returnee	Evacuees in Lifted Areas	Evacuees in Banned Areas
No. at risk, *n*	6505	448	1062	4498	10,279	836	2145	7226
Diabetes, *n*	450	30	59	402	425	33	59	364
Age-adjusted OR (95% CI)	Ref.	1.06 (0.72–1.55)	1.01 (0.76–1.34)	1.46 (1.27–1.68) ***	Ref.	1.06 (0.74–1.52)	0.94 (0.71–1.24)	1.41 (1.22–1.63) ***
Multivariable OR (95% CI) ^§^	Ref.	1.03 (0.70–1.52)	1.03 (0.77–1.37)	1.33 (1.15–1.55) ***	Ref.	1.06 (0.73–1.52)	0.97 (0.73–1.28)	1.38 (1.19–1.61) ***
Multivariable OR (95% CI) ^§§^	Ref.	1.02 (0.69–1.51)	1.02 (0.77–1.37)	1.33 (1.15–1.54) ***	Ref.	1.05 (0.73–1.52)	0.96 (0.72–1.28)	1.38 (1.18–1.60) ***
No. at risk, *n*	3259	245	718	2473	6108	563	1699	4863
Hypertension, *n*	789	51	122	589	1039	95	145	779
Age-adjusted OR (95% CI)	Ref.	0.95 (0.69–1.33)	0.90 (0.72–1.12)	1.13 (0.99–1.28) *	Ref.	1.16 (0.91–1.47)	0.81 (0.67–0.99)	1.21 (1.08–1.34) **
Multivariable OR (95% CI) ^§^	Ref.	0.92 (0.66–1.29)	0.93 (0.74–1.16)	1.08 (0.95–1.23)	Ref.	1.13 (0.89–1.45)	0.84 (0.69–1.02)	1.20 (1.08–1.35) **
Multivariable OR (95% CI) ^§§^	Ref.	0.92 (0.66–1.28)	0.93 (0.74–1.16)	1.08 (0.94–1.23)	Ref.	1.13 (0.89–1.45)	0.84 (0.69–1.02)	1.20 (1.08–1.35) **
No. at risk, *n*	3612	225	627	2258	5016	392	1404	3839
Dyslipidemia, *n*	890	50	157	698	1210	102	264	990
Age-adjusted OR (95% CI)	Ref.	0.87 (0.63–1.20)	1.01 (0.83–1.23)	1.36 (1.21–1.53) ***	Ref.	1.28 (1.01–1.62)	0.97 (0.83–1.14)	1.24 (1.12–1.37) ***
Multivariable OR (95% CI) ^§^	Ref.	0.85 (0.61–1.18)	1.01 (0.82–1.23)	1.31 (1.16–1.48) ***	Ref.	1.24 (0.98–1.58)	0.96 (0.82–1.13)	1.21 (1.09–1.34) ***
Multivariable OR (95% CI) ^§§^	Ref.	0.85 (0.61–1.18)	1.01 (0.83–1.24)	1.31 (1.16–1.48) ***	Ref.	1.24 (0.97–1.57)	0.96 (0.82–1.23)	1.20 (1.09–1.33) ***

* *p* < 0.05; ** *p* < 0.01; *** *p* < 0.001. ^§^ Adjust for age, body mass index, smoking status, alcohol consumption, sports time, sleep quality, education level, and change of job. ^§§^ Adjusted further for psychological distress.

## Data Availability

The datasets analyzed during the present study are not publicly avail-able because the data from the Fukushima Health Management Survey belongs to the government of Fukushima Prefecture and can only be used within the organization.

## Supplementary Materials

The following supporting information can be downloaded at: https://www.mdpi.com/article/10.3390/ijerph19095661/s1, Table S1: Global Moran index of the spatial distribution of the prevalence of lifestyle-related diseases by administrative division among examinees.
