# Supplementary material for: The Associations between Evacuation Status and Lifestyle-Related Diseases in Fukushima after the Great East Japan Earthquake: The Fukushima Health Management Survey
Source: Int J Environ Res Public Health. 2022 May 6;19(9):5661. doi: 10.3390/ijerph19095661 (PMC9105675; doi:10.3390/ijerph19095661)
Supplement: Supplementary file 1 [file ijerph-19-05661-s001.zip › Table S1.pdf]

Table S1. Global Moran index of the spatial distribution of the prevalence of lifestyle-related diseases by administrative division among examinees

	P	*Z* score	Moran's index
Diabetes	0.07	1.78	0.17
Hypertension	0.04	2.04	0.16
Dyslipidemia	0.01	4.25	0.34
